# Clustering and Correlates of Multiple Health Behaviours in 9–10 Year Old Children

**DOI:** 10.1371/journal.pone.0099498

**Published:** 2014-06-12

**Authors:** Leonie K. Elsenburg, Eva Corpeleijn, Esther M. F. van Sluijs, Andrew J. Atkin

**Affiliations:** 1 Department of Epidemiology, University of Groningen, University Medical Centre Groningen, Groningen, Netherlands; 2 Medical Research Council, Epidemiology Unit, University of Cambridge School of Clinical Medicine, Cambridge, United Kingdom; 3 UKCRC Centre for Diet and Activity Research (CEDAR), MRC Epidemiology Unit, University of Cambridge School of Clinical Medicine, Cambridge, United Kingdom; Pennington Biomedical Research Center, United States of America

## Abstract

**Background:**

Sleep, physical activity, screen time and dietary behaviours influence health during childhood, but few studies have looked at all of these behaviours simultaneously and previous research has relied predominantly on self- or proxy-reports of physical activity and food frequency questionnaires for the assessment of diet.

**Purpose:**

To assess the prevalence and clustering of health behaviours and examine the socio-demographic characteristics of children that fail to meet multiple health behaviour guidelines.

**Methods:**

Data are from the Sport, Physical activity and Eating behaviour: Environmental Determinants in Young people (SPEEDY) study. Participants (n = 1472, 42.9% male) were dichotomized based on whether or not they met public health guidelines for accelerometer-assessed physical activity, diet-diary assessed fruit/vegetable intake and fat/non-milk extrinsic sugar (NMES) intake, and self-reported screen time and sleep duration. Behavioural clustering was assessed using an observed over expected ratio (O/E). Socio-demographic characteristics of participants that failed to meet multiple health behaviour guidelines were examined using ordinal logistic regression. Data were analysed in 2013.

**Results:**

83.3% of children failed to meet guidelines for two or more health behaviours. The O/E ratio for two behavioural combinations significantly exceeded 1, both of which featured high screen time, insufficient fruit/vegetable consumption and excessive fat/NMES intake. Children who were older (Proportional odds ratio (95% confidence interval): 1.69 (1.21,2.37)) and those that attended a school with a physical activity or diet-related policy (1.28 (1.01,1.62)) were more likely to have a poor health behaviour profile. Girls (0.80 (0.64,0.99)), participants with siblings (0.76 (0.61,0.94)) and those with more highly educated parents (0.73 (0.56,0.94)) were less likely to have a poor health behaviour profile.

**Conclusions:**

A substantial proportion of children failed to meet guidelines for multiple health behaviours and there was evidence of clustering of screen viewing and unhealthy dietary behaviours. Sub-groups at greatest risk may be targeted for intervention.

## Introduction

Modifiable lifestyle behaviours, including poor diet, physical inactivity, and excessive sedentary behaviour, contribute to non-communicable disease morbidity and mortality [1], [2]. Whilst chronic disease emerges in adulthood, disease precursors and behaviour patterns are established during childhood, suggesting that surveillance and promotion of health behaviours should start early in life [3]–[5]. In young people, physical activity, dietary patterns, sleep and sedentary behaviours have been associated with overweight and obesity, cardio-metabolic risk and mental ill-health, though evidence from prospective and experimental research is limited in some cases [6]–[10]. Despite varying strength of the underlying evidence, public health guidelines have been established to indicate optimal levels of these behaviours during childhood [11]–[15].

Numerous studies have described the prevalence and distribution of single health behaviours in young people, but far fewer have examined the patterning of multiple health behaviours [16]–[23]. Understanding behavioural clustering, the co-occurrence of behaviours at a level that exceeds their combined individual prevalences [24], [25], is important because disease risk may increase synergistically when multiple risk factors occur simultaneously [26]. Moreover, the identification of populations that fail to meet guidelines for multiple health behaviours may enable appropriate targeting of intervention programmes. Evidence that health behaviour patterns established in childhood persist into adulthood supports the identification of health behaviour profiles early in life [5], particularly in the years preceding adolescence which may mark a transitional phase in young people's health behaviour.

In Australian adolescents, Hardy et al. [27] reported clustering of sedentary, physically active and dietary behaviours, particularly amongst participants from low income households, based upon comparison of observed to expected prevalences. Similarly, Dumith et al. [28] found that smoking, diet and physical activity appeared to cluster amongst Brazilian adolescents, with those participants who were older, female, and from households with fewer resources more likely to report multiple health risk behaviours. To date, research into health behaviour clustering in young people has focussed mostly on adolescents or has typically relied on relatively crude instruments to assess behaviour, such as self-reports of physical activity or food frequency questionnaires to assess diet [16]–[19], [27]–[30]. Moreover, existing studies have often used sample-specific thresholds for determining optimal behaviour levels, which may have limited relevance or validity at population level. To further understanding of the clustering of obesogenic behaviours, we aimed to examine the prevalence and clustering of multiple health behaviours in a large population-based sample of children, using accelerometry to assess physical activity, a 4-day food diary to ascertain diet and questionnaires to obtain screen and sleep time. Additionally, we investigated the socio-demographic characteristics of children that failed to meet multiple health behaviour guidelines.

## Methods and Materials

### Design and procedure

Data are from the Sport, Physical activity and Eating behaviour: Environmental Determinants in Young people (SPEEDY) study, a longitudinal cohort examining factors associated with physical activity, sedentary behaviour and diet in children from the county of Norfolk, UK. Ethical approval for the SPEEDY study was obtained from the University of East Anglia research ethics committee. Full details of participant recruitment and procedures for data collection are described elsewhere [31]. Of the 157 schools approached to participate in SPEEDY, 92 were visited for measurement. At participating schools, all children in school year 5 (Age 9-10 years; N = 3619) and their parents were sent an invitation pack. In total, 2064 children provided parental consent and were included in data collection (response rate 57.0%). Data collection took place during the school term, between April-July 2007. Trained research assistants visited schools to take physical measurements, administer child questionnaires, fit accelerometers, and distribute a home pack (containing an accelerometer diary, instruction sheet, parent/guardian questionnaire, and food diary). Participants were requested to return the home pack one week later. Height (Leicester height measures; Chasmors Ltd, Leicester, UK), weight in light clothing and foot-to-foot bio-impedance (TBF-300A; Tanita, Tokyo, Japan) were assessed by trained research assistants. Fat free mass, fat mass and percentage body fat were calculated using validated equations [32]. Body mass index (BMI) was calculated as weight (kg)/height^2^ (m^2^) and weight status determined using international cut-off values [33].

### Physical activity

Physical activity was assessed objectively using the Actigraph accelerometer (GT1M; Actigraph, Pensacola, FL), which has demonstrated accuracy for the assessment of energy expenditure in children under free living conditions [34]. Children were asked to wear the monitor during waking hours for 7 days and to remove it while bathing, showering, and swimming. The monitors were set to record at 5-second epochs. Accelerometer data were analysed using a batch processing program (MAHUffe; www.mrc-epid.cam.ac.uk/Research/Programmes/Programme_5/InDepth/Programme%205_Disclaimer.html) and data recorded after 11PM and before 6AM were removed. Periods of ≥10 minutes of consecutive zero counts, days with <500 minutes of recording and participants with <3 days of recording were excluded from analyses. A threshold of 2000 counts/min was used to define moderate to vigorous intensity physical activity (MVPA), as previous research has shown both ≥1770 counts/min and ≥2296 counts/min exhibit excellent classification accuracy for MVPA in this age group [35]. Public health guidelines state that children should accumulate at least 60 minutes of MVPA each day [12].

### Screen time

Time spent watching television (including video/DVD; 1 week test-retest reliability ICC = 0.93), using a computer (including the internet; 1 week test-retest reliability ICC = 0.53) and playing computer games (1 week test-retest reliability ICC = 0.75) was assessed using the Youth Physical Activity Questionnaire (YPAQ; [36]), which is based on the Children's Leisure Activities Study Survey (CLASS; [37]). Separately for weekdays and weekend days, participants indicated the number of days they had engaged in these behaviours and the average duration of participation per episode in the past 7 days. Weighted mean duration of each behaviour per day ((5*weekday+2*weekend)/7) was derived and summed to provide a measure of screen time. Children accumulating an average of less than 2 hours of screen viewing per day were coded as meeting the guidelines [11].

### Sleep time

Weighted average daily sleep duration was derived from children's self-reported usual time of going to bed and getting up on week and weekend days, respectively. Based upon age-specific guidelines from the National Sleep Foundation, sufficient sleep was defined as ≥10.5 hours/night [13].

### Dietary behaviour

Two dietary constructs were examined: 1) consumption of fruits and vegetables and, 2) intake of total dietary fat and non-milk extrinsic sugars (NMES). Food intake was recorded using a four-day unweighed food and drink diary where children, with assistance from their parents, were asked to record everything they ate and drank over a four-day period (including 2 weekend days). This method has previously been used and validated with children aged 9–10 years, correlations between observed and reported nutrient and energy intake ranged from 0.78 to 0.94 [38]. Estimated weights of portions were then calculated using published values, including those specific to children [39], [40]. Based upon a recommended portion size of 40 grams for school age children [41] and public health guidelines of 5 portions per day [15], achievement of recommendations for fruit and vegetable intake was defined as ≥200 grams/day.

For total dietary fat and NMES, nutrient adequacy ratios (NAR) were derived using the following formula:
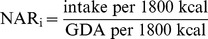



An NAR of >1 indicates that intake exceeds the recommended level relative to the adjusted energy intake. For total dietary fat and NMES, guideline daily amounts (GDA) of 70 grams/day and 50 grams/day are recommended per 1800 kcal energy intake [42]. The mean adequacy ratio (MAR) was derived as a simple average of NAR for the 2 dietary components. In line with recommendations that total fat and NMES intake should be limited in the diet, a MAR ≤1 was judged to be indicative of healthy dietary practice.

### Demographic information

Participants self-reported their age and sex. Parental education (‘GCSE or lower (General Certificate of Secondary Education)’, ‘up to A-level (General Certificate of Education Advanced)’ and ‘higher education’) was used as a proxy for socio-economic position (SEP). Postal code was used to determine urban/rural location of participants home. Four density profiles were collapsed into a dichotomous variable (city, town and fringe: urban; hamlets and isolated dwellings/villages: rural) [43].

### School policy

Head teachers reported separately whether or not the school had a policy related to physical activity or diet. Many interventions to promote healthy lifestyles in young people are delivered through the school, thus it was of interest to examine the association between health-related school policies and children's health behaviour profile [44].

### Statistical analysis

Analyses were conducted using Stata (Stata, College Station, TX) in 2013. Sample characteristics were summarised using descriptive statistics. As appropriate, t-tests and X^2^ tests were used to examine gender differences in demographic, anthropometric and behavioural variables.

Behavioural clustering was determined by the ratio of the observed to the expected prevalence of failing to meet guidelines for two to five health behaviours simultaneously, as described previously [25]. Separately for all behaviour combinations, observed prevalences were calculated as the number of participants that did or did not meet guideline levels for each health behaviour divided by the total number of participants (e.g. the proportion of children that had low physical activity and high screen time but had sufficient sleep, fruit and vegetable intake and MAR less than 1). The expected prevalence for single behaviours was calculated as the proportion of participants not meeting a specific guideline multiplied by the proportion of participants that met the guideline for all remaining behaviours (e.g. the proportion of children that had high screen time multiplied by the proportion that had adequate physical activity, the proportion that had enough sleep, the proportion that had sufficient fruit and vegetable intake and the proportion with MAR less than 1). The expected prevalence for multiple health behaviours was calculated by multiplying the proportion of participants that did not meet guideline levels for a specific set of behaviours and the proportion that met guideline levels for all remaining behaviours. A ratio of the observed over the expected prevalence (O/E) was then calculated to examine whether health behaviours co-occurred at a higher (or lower) rate than would be expected if there was no association between behaviours. Ninety five percent confidence intervals were calculated using bootstrap techniques. Observed over expected ratios >1 are indicative of clustering.

A health behaviour risk score (range 0–5) was derived as the number of unmet health behaviour guidelines, and used as the outcome variable in ordinal logistic regression models examining the socio-demographic characteristics of children displaying a higher number of unmet guidelines. Proportional odds ratios indicate the effect of a 1 unit increase in the exposure on the odds of having a higher health behaviour risk score relative to all combined lower health behaviour risk scores, controlling for other variables in the model. Robust standard errors (Huber-White Sandwich Estimator) were used to account for potential non-independence of participants resulting from school-based recruitment. The Brant test was used to test for violations of the proportional odds assumption. Candidate correlates with p≤0.25 in bivariate analysis were retained for inclusion in a multivariable model. Sex, body fat percentage and parental education were retained in the multivariable model irrespective of their significance level. A p-value of <0.05 was used to indicate statistical significance in the multivariable model.

## Results

After exclusion of participants with incomplete data, 1472 (71.3% of those included in data collection) children were included in the analyses. Participant characteristics are presented in Table 1. Analyses comparing the analytical sample with the excluded children revealed no differences in age or body fat percentage. The analytical sample did, however, contain a higher proportion of girls (57.1% vs. 50.3%, *p* = 0.005), had parents with a higher overall educational attainment (*p* = 0.005), had a higher fruit and vegetable intake (197.7 vs. 182.4 g/day, *p* = 0.02) and exceeded recommended limits of total fat and NMES to a greater degree (MAR = 1.19 vs. 1.14, *p*<0.001) than the excluded children.

**Table 1:** Participant characteristics (mean (SD) unless stated otherwise; n = 1472).

	All		Boys		Girls		P
**n (%)**	1472		632	(42.9)	840	(57.1)	
**Age, years**	10.3	(0.3)	10.2	(0.3)	10.3	(0.3)	0.52
**Body fat %**	30.7	(7.9)	27.2	(7.4)	33.2	(7.2)	<0.01
**BMI**	18.3	(3.2)	17.9	(2.8)	18.5	(3.4)	<0.01
**Weight status, %**							<0.01
Overweight	18.1		16.0		19.8		
Obese	5.3		3.8		6.4		
**Parent education, %**							0.02
GCSE or lower	36.8		32.9		39.8		
Up to A level	42.7		44.6		41.2		
Higher education	20.5		22.5		19.1		
**MVPA, mins/day**	73.7	(24.8)	84.2	(25.8)	65.9	(20.7)	<0.01
<60 mins/day, %	31.8		18.8		41.6		<0.01
**Screen time, mins/day**	138.5	(118.0)	168.0	(129.7)	116.3	(103.1)	<0.01
>120 mins/day, %	43.4		53.6		35.7		<0.01
**Sleep time, mins/day**	631.9	(44.9)	622.8	(48.0)	638.7	(41.1)	<0.01
<10.5 hours/night, %	44.8		51.7		39.5		<0.01
**Fruit and vegetable intake, g/day**	197.7	(115.8)	191.6	(113.7)	202.2	(117.2)	0.08
<200 g/day, %	56.1		57.4		55.1		0.38
**MAR**	1.2	(0.23)	1.2	(0.24)	1.2	(0.22)	0.07
MAR >1, %	78.7		79.8		78.0		0.41

P-value is for difference between boys and girls.

BMI, Body mass index; GCSE, General Certificate of Secondary Education; A level, General Certificate of Education Advanced level; MVPA, moderate-to-vigorous intensity physical activity; MAR, mean adequacy ratio (total dietary fat and non-milk extrinsic sugar).

Table 1 also shows the prevalence of not meeting individual health behaviour guidelines. Seventy-eight percent of participants exceeded the recommended intake of fat and NMES (MAR>1) and 56.1% failed to consume sufficient fruits and vegetables. Insufficient physical activity was more prevalent in girls than boys, but boys were more likely to exceed screen time recommendations and get insufficient sleep. The prevalence of not meeting 0, 1, 2, 3, 4, and 5 health behaviour guidelines was 2.5%, 14.2%, 32.5%, 30.6%, 17.1% and 3.1%.

### Clustering of health behaviours

Thirty-two possible combinations of the five health behaviours were examined (Table 2). Overall, observed over expected ratios were close to 1 and ranged from 0.54 to 1.48. The 4-risk behaviour combination of excessive screen time, insufficient fruit and vegetable consumption, and MAR greater than 1, in combination with either low physical activity (O/E (95% CI): 1.31 (1.04, 1.59)) or inadequate sleep (1.22 (1.03, 1.41) occurred more frequently than expected. Four health behaviour combinations, wherein guidelines were not met for 2 or 3 health behaviours, occurred less frequently than expected.

**Table 2:** Observed and expected prevalence of not meeting health behaviour guidelines, individually and in combination.

No. of unmet guidelines	Low physical activity	High screen time	Low sleep time	Low fruit and vegetable intake	High MAR	O (%)	E (%)	O/E (95% CI)	
**5**	+	+	+	+	+	3.06	2.73	1.12	(0.82, 1.42)
**4**	+	+	+	+	-	0.75	0.74	1.01	(0.42, 1.61)
	+	+	+	-	+	1.90	2.14	0.89	(0.59, 1.19)
	+	+	-	+	+	4.42	3.37	**1.31**	**(1.04, 1.59)**
	+	-	+	+	+	2.92	3.56	0.82	(0.59, 1.05)
	-	+	+	+	+	7.13	5.86	**1.22**	**(1.03, 1.41)**
**3**	+	-	+	+	-	0.82	0.96	0.85	(0.39, 1.31)
	+	+	-	+	-	0.75	0.91	0.82	(0.38, 1.27)
	+	-	-	+	+	5.10	4.39	1.16	(0.94, 1.38)
	+	+	+	-	-	0.34	0.58	0.59	(0.06, 1.12)
	+	-	+	-	+	1.83	2.78	**0.66**	**(0.43, 0.89)**
	+	+	-	-	+	1.97	2.63	**0.75**	**(0.50, 0.99)**
	-	+	+	+	-	1.90	1.58	1.20	(0.80, 1.60)
	-	-	+	+	+	7.00	7.63	0.92	(0.78, 1.06)
	-	+	-	+	+	5.64	7.23	**0.78**	**(0.64, 0.92)**
	-	+	+	-	+	5.30	4.58	1.16	(0.94, 1.38)
**2**	+	-	-	+	-	1.56	1.19	1.32	(0.81, 1.82)
	+	-	+	-	-	0.41	0.75	**0.54**	**(0.13, 0.96)**
	+	+	-	-	-	0.61	0.71	0.86	(0.32, 1.40)
	+	-	-	-	+	4.28	3.43	1.25	(0.99, 1.50)
	-	-	+	+	-	1.90	2.06	0.92	(0.60, 1.24)
	-	+	-	+	-	1.56	1.95	0.80	(0.50, 1.11)
	-	-	-	+	+	8.76	9.42	0.93	(0.80, 1.06)
	-	+	+	-	-	1.83	1.24	1.48	(0.97, 2.00)
	-	-	+	-	+	6.39	5.97	1.07	(0.89, 1.25)
	-	+	-	-	+	5.16	5.65	0.91	(0.74, 1.09)
**1**	+	-	-	-	-	1.09	0.93	1.17	(0.63, 1.71)
	-	-	-	+	-	2.85	2.54	1.12	(0.82, 1.42)
	-	-	+	-	-	1.29	1.61	0.80	(0.46, 1.14)
	-	+	-	-	-	1.09	1.53	0.71	(0.39, 1.04)
	-	-	-	-	+	7.88	7.37	1.07	(0.91, 1.23)
**0**	-	-	-	-	-	2.51	1.99	1.26	(0.90, 1.63)

O, observed prevalence; E, expected prevalence; 95% CI, 95% confidence interval; +, guideline not met; -, guideline met; MAR, mean adequacy ratio.

### Correlates of health behaviour risk score

Table 3 presents the bivariate and multivariate associations. The proportional odds ratio indicates the effect of a 1 unit increase in the exposure on the odds of having a higher health behaviour risk score relative to all combined lower health behaviour risk scores, controlling for other variables in the model. In the multivariate analysis, children who were older and those that attended a school with a physical activity or diet-related policy were more likely to have a poor health behaviour risk score (more unmet guidelines). Girls, participants with siblings and those with more highly educated parents were less likely to have a poor health behaviour risk score. The distribution of health behaviour risk scores by socio-demographic characteristics are presented in Table 4.

**Table 3:** Association of anthropometric and socio-demographic characteristics with health behaviour risk score (No. of unmet guidelines) (n = 1472).

	Bivariate analysis	Adjusted analysis
	POR (95% CI)	POR (95% CI)
**Age**	1.74 (1.24, 2.45)**	1.69 (1.21, 2.37)**
**Body fat %**	1.00 (0.99, 1.01)	1.00 (0.99, 1.01)
**Sex (ref: male)**	0.83 (0.68, 1.01)	0.80 (0.64, 0.99)**
**Parent education**		
GCSE or lower	ref	ref
Up to A level	0.94 (0.77, 1.14)	0.94 (0.77, 1.15)
Higher education	0.72 (0.55, 0.93)**	0.73 (0.56, 0.94)**
**Home location**		
Rural	ref	ref
Urban	1.12 (0.94, 1.33)*	1.08 (0.91, 1.29)
**Family structure**		
1 parent	ref	Ref
2 parents	0.81 (0.64, 1.02)*	0.89 (0.70, 1.12)
**Siblings**		
None	ref	ref
One or more	0.72 (0.58, 0.90)**	0.76 (0.61, 0.94)**
**School policy**		
No	ref	ref
Yes	1.31 (1.02, 1.68)**	1.28 (1.01, 1.62)**

* p≤0.25.

**p≤0.05.

POR, proportional odds ratio; CI, confidence interval; ref, reference group.

GCSE, General Certificate of Secondary Education; A level, General Certificate of Education Advanced level.

**Table 4:** Health behaviour risk score (No. of unmet guidelines) by socio-demographic and anthropometric characteristics (n (%) unless stated otherwise).

	Health behaviour risk score											
	0		1		2		3		4		5	
**All**	37	(2.5)	209	(14.2)	478	(32.5)	451	(30.6)	252	(17.1)	45	(3.1)
**Age, mean (SD)**	10.2	(0.3)	10.2	(0.3)	10.2	(0.3)	10.3	(0.3)	10.3	(0.3)	10.2	(0.3)
**Body fat %, mean (SD)**	32.1	(8.1)	30.6	(7.1)	30.5	(7.6)	30.5	(8.0)	30.9	(8.4)	31.8	(8.8)
**Sex**												
Boy	11	(29.7)	88	(42.1)	191	(40.0)	206	(45.7)	116	(46.0)	20	(44.4)
Girl	26	(70.3)	121	(57.9)	287	(60.0)	245	(54.3)	136	(54.0)	25	(55.6)
**Parent education**												
GCSE or lower	9	(24.3)	64	(30.6)	185	(38.7)	170	(37.7)	98	(38.9)	16	(35.6)
Up to A level	19	(51.4)	93	(44.5)	188	(39.3)	195	(43.2)	109	(43.3)	24	(53.3)
Higher education	9	(24.3)	52	(24.9)	105	(22.0)	86	(19.1)	45	(17.9)	5	(11.1)
**Home location**												
Rural	16	(43.2)	70	(33.5)	174	(36.4)	151	(33.5)	80	(31.7)	15	(33.3)
Urban	21	(56.8)	139	(66.5)	304	(63.6)	300	(66.5)	172	(68.3)	30	(66.7)
**Family structure**												
1 parent	4	(10.8)	25	(12.0)	83	(17.4)	69	(15.3)	53	(21.0)	5	(11.1)
2 parents	33	(89.2)	184	(88.0)	395	(82.6)	382	(84.7)	199	(79.0)	40	(88.9)
**Siblings**												
None	3	(8.1)	27	(12.9)	80	(16.7)	78	(17.3)	53	(21.0)	10	(22.2)
One or more	34	(91.9)	182	(87.1)	398	(83.3)	373	(82.7)	199	(79.0)	35	(77.8)
**School policy**												
Yes	27	(73.0)	168	(80.4)	394	(82.4)	380	(84.3)	213	(84.5)	41	(91.1)
No	10	(27.0)	41	(19.6)	84	(17.6)	71	(15.7)	39	(15.5)	4	(8.9)

GCSE: General Certificate of Secondary Education; A level: General Certificate of Education Advanced level.

## Discussion

We examined the prevalence, clustering and correlates of multiple health behaviours in a large population based sample of primary school children from the UK. Analyses revealed that the majority of children failed to meet guidelines for 1 or more health behaviour(s), and that high levels of screen viewing and poor dietary practices may cluster in this population. Children that failed to meet multiple health behaviour guidelines were more likely to be male, older, have parents with lower educational attainment and have no siblings.

With the exception of MVPA in boys, at least 30% of participants in the current study failed to meet guidelines for each of the 5 health behaviours, with fruit and vegetable intake and MAR least likely to be reported at recommended levels. Observed sex differences in MVPA, screen time and fruit and vegetable intake are consistent with existing literature [20]–[23]. There is some evidence of longer habitual sleep duration in girls compared to boys, but sex differences in children's sleep have not been established definitively [45]–[47]. Whilst overall trends in health behaviour patterns are broadly consistent with previous research, the direct comparison of prevalence estimates is limited by contrasting approaches to behavioural assessment and the operationalization of public health guidelines [48]. Nonetheless, the evidence appears sufficiently robust to warrant continued efforts to understand the determinants of these behaviours and supports the development of interventions to promote optimal levels.

Two health behaviour combinations occurred significantly more often than expected, both of which were characterised by high levels of screen viewing, low fruit and vegetable consumption and MAR greater than 1. Concurrently, the behavioural cluster in which these three behaviours were performed at recommended levels occurred less frequently than expected. Results are consistent with existing evidence linking screen-based sedentary behaviour with poor dietary practices [49] and with findings from Hardy et al. who reported an observed/expected ratio of 2.3 (95% CI: 1.3, 3.9) for high screen time, low fruit and vegetable intake, and high soft-drink and snacking in adolescent girls [27]. They are also in line with previous studies investigating clustering in children in which clusters characterized by high screen time and poor dietary patterns were identified [16]–[19]. The co-occurrence of excessive screen time and poor diet may be due to these behaviours having shared determinants or it may be that one behaviour precedes and precipitates the other; due to the cross-sectional nature of the current study, however, temporal sequence cannot be established. It has been hypothesised that unhealthy dietary behaviours may mediate the association between screen viewing and obesity in young people, and experimental evidence indicates that limiting screen time in children can lead to reductions in BMI z-score via lowered energy intake without concomitant increases in physical activity [50]. The utility of interventions targeting multiple health behaviours for behaviour change and weight management warrants further investigation.

We identified a number of socio-demographic factors that were associated with not meeting multiple health behaviour guidelines, including older age, being an only child and having parents with low educational attainment. Given the relatively narrow age range of the current sample, it was unexpected that increasing age was associated with a poorer health behaviour profile, but this is consistent with previous research [51] and suggests that clustered behaviour patterns established in childhood may persist into adolescence. Also consistent with our findings, higher numbers of behavioural risk factors have been identified amongst young people living in low income households, those with lower parental education or those who scored lower on an index of household assets [27]–[29]. Collectively, these studies highlight socio-economic disparities in health behaviour during childhood that may account, in part, for health and well-being inequalities observed later in life [52]. We also found that participants who attended a school with policies related to physical activity or diet were more likely to have a higher health behaviour risk score (more unmet guidelines). Given the cross-sectional nature of this analysis, it is not possible to ascertain whether this association is the result of such policies adversely impacting upon children's health behaviour or whether head-teachers introduced diet and activity related policies in response to poor health behaviour profiles amongst pupils, but the latter seems the more plausible explanation. Unfortunately, our headteacher questionnaire did not include assessment of when policies were introduced, their content or implementation. This information, combined with a longitudinal design, would be valuable in future studies that examine the complex association between policies and behaviour in children [53].

Strengths of the current study include the collection of data from a large population based sample and the use of accelerometers to measure physical activity objectively. In addition, we conducted dietary assessment by food diary, which is less susceptible to bias than food frequency questionnaires or recall instruments [54]. Behavioural information was collected using independent sources of information, reducing the likelihood of spurious associations due to correlated error. A limitation is that the SPEEDY sample was recruited from a relatively small geographic region with limited ethnic heterogeneity relative to the broader UK population. There was also evidence of some selective drop-out, by sex and SEP, which may limit generalizability of findings. We acknowledge alternative statistical approaches to the analysis of multiple behaviours and behavioural clustering [24], for example cluster analysis, but felt the current approach has greater public health relevance as it enabled us to characterize our sample relative to established public health guidelines rather than on the basis of within sample variability.

### Conclusion

In conclusion, this study revealed that a substantial proportion of children aged 9-10 years failed to meet guidelines for 1 or more health behaviours, and that high levels of screen viewing and poor dietary practices may cluster in this population. Based upon the behaviours selected for the current study, children at greatest risk for not meeting multiple health behaviour guidelines, and potential targets for tailored interventions, include boys, those who are older or without siblings, and those with parents of lower educational attainment.
